# What do register-based studies tell us about migrant mental health? A scoping review

**DOI:** 10.1186/s13643-017-0463-1

**Published:** 2017-04-11

**Authors:** Kishan Patel, Anne Kouvonen, Ciara Close, Ari Väänänen, Dermot O’Reilly, Michael Donnelly

**Affiliations:** 1 Administrative Data Research Centre (Northern Ireland), Centre for Public Health, Queen’s University Belfast, Belfast, UK; 2 Department of Social Research, University of Helsinki, Helsinki, Finland; 3 SWPS University of Social Sciences and Humanities in Wroclaw, Wroclaw, Poland; 4 UKCRC Centre of Excellence for Public Health, Queen’s University Belfast, Belfast, UK; 5 Finnish Institute of Occupational Health, Helsinki, Finland

**Keywords:** Migration, Migrants, Immigrants, Mental health, Register data, Record-linkage, Scoping review

## Abstract

**Background:**

Previous studies investigating the mental health of migrants have shown mixed results. The increased availability of register data has led to a growing number of register-based studies in this research area. This is the first scoping review on the use of registry and record-linkage data to examine the mental health of migrant populations. The aim of this scoping review is to investigate the topics covered and to assess the results yielded from these studies.

**Methods:**

We used a scoping review methodology to search MedLine, PubMed, PsychINFO, Web of Science, and SCOPUS for all register-based studies on the mental health of migrants. Two reviewers screened all papers, independently, using iteratively applied inclusion and exclusion criteria. Using gradually broadening inclusion and exclusion criteria for maximum “scope,” newly published criteria developed to appraise the methodological quality of record-linkage studies were applied to eligible papers and data were extracted in a charting exercise.

**Results:**

A total of 1309 papers were screened and appraised, 51 of which met the eligibility and quality criteria and were included in the review. This review identified four major domains of register-based research within the topic of migrant mental health: rates and risks of psychiatric disorders, rates and risks of suicide mortality, the use of psychotropic drugs, and health service utilisation and mental health-related hospitalisation rates. We found that whilst migrants can be at an increased risk of developing psychotic disorders and suicide mortality, they are less likely to use psychotropic medication and mental health-related services.

**Conclusions:**

This review systematically charts the register-based studies on migrants’ mental health for the first time. It shows the main topics and gaps in knowledge in this research domain, discusses the disadvantages of register-based studies, and suggests new directions for forthcoming studies.

**Electronic supplementary material:**

The online version of this article (doi:10.1186/s13643-017-0463-1) contains supplementary material, which is available to authorized users.

## Background

According to “the healthy migrant effect,” immigrants, including refugees, individuals who are born outside their current country of residence tend to be among the healthiest groups in their original populations and to be healthier, on average, than the settled majority in their new countries [1]. However, this effect seems to become less pronounced soon after migration [2]. Research worldwide has shown mixed results with regards to the prevalence of mental ill-health for migrants when compared to their settled majority populations [3].

Due to improved availability of administrative record data, register-based studies have become increasingly common in epidemiology and public health [4]. Registers tend to be organised into administrative, disease, reimbursement, and quality registers and then linked with population registers to address a range of questions about various health conditions. Register data are not collected specifically for research purposes [5], and their parameters are determined by decisions regarding the function and process of producing the data. Also, the use of registries for research purposes is influenced by the data available and the knowledge of the researcher. Understanding the variables and the strengths and limitations of registers provides opportunities to improve the methodology. In addition, most health records are collected for administration purposes and, so, variables may change over time as social and health policies change. Therefore, there is a need for researchers to be aware of these changes in order to avoid making incorrect conclusions.

However, a number of studies (particularly in Nordic countries) have championed their validity in terms of assessing public health matters. In addition, their records have been assessed as reasonably complete and they can be widely linked using national ID numbers, and, therefore, they are particularly useful for record-linkage studies. For example, records of short-term care in Swedish hospitals have been estimated to be under-reported by less than 2%. Diagnostic categories in Finnish and Swedish inpatient registers have been assessed as highly valid [6]. The estimated coding error in the Swedish cause-of-death register was only 3% [7]. Similarly, any entries into the Finnish cause-of-death register have to pass a routine validation test [8]. Finally, the coverage of the Danish cause-of-death register, the Finnish hospital discharge register, and the Swedish hospital discharge register have been assessed as highly complete [9]. The large number of high-quality studies in Nordic countries, along with the area’s long-standing use of national registers, is particularly relevant as it is likely that they will form a large part of our scope.

The quality and availability of these datasets offer unique opportunities to investigate migrant mental health at population level. For example, Cantor-Graae [10] and Pederson [11] used national registers to conduct studies concerning populations of 1.06 and 1.1 million, respectively. It is possible also to study specific sub-groups within migrant populations using these datasets. Examples include, among others, studies of refugees and asylum seekers [12, 13], distinct first- and second-generation migrant groups [14–16], migrants of specific ages [10, 17], and migrants of certain ethnic backgrounds [18, 19]. However, whilst many studies have investigated the mental health of migrants using register-based data, to our best knowledge, no review of these studies has been conducted. Due to the heterogeneous nature of the studies conducted in the topic area, we decided to follow a scoping review methodology. A scoping review allows a broader research question to be addressed and captures a wider range of studies than a systematic review [20]. A scoping review can also be used to identify areas that are likely to benefit from a further systematic review.

## Methods

In designing this scoping review, we followed the methods defined by Armstrong et al. [20], Daudt et al. [21], and Arksey and O’Malley [22]. Our review was conducted in five broad stages, each of which is outlined below.

### Stage 1: identifying the research questions

The framework outlined by Arksey and O’Malley [22] suggests an iterative approach when formulating a research question. Our increasing familiarity with the available literature led us to specify the following guiding questions in the area of migrant mental health:

#### Primary questions

1. What are the key topics/issues covered by studies using register-based data?
  Is there a focus on certain disorders or are disorders studied in equal numbers? What else is being studied?
2. What are the topics that are most amenable with register-based data?
  Are there certain areas of mental health that can be explored more fully than others?
3. What are the reported advantages and disadvantages of register-based studies?
  Why are register-based studies useful, according to the authors of individual papers?
  What problems did those authors encounter?

We also developed two subsidiary questions that could not be answered as fully as our primary questions, due to the nature of our scoping study:

#### Subsidiary questions

4. What are the key gaps in the existing knowledge?
  Which areas need more research?
  Are there reasons for certain areas being under-researched?
5. How well do register-based studies answer similar questions compared to other data types?
  Do they offer any specific advantages over e.g., surveys or interviews?

For each question, the overarching questions were developed to guide initial thinking, and the sub-questions were added to clarify the parameters of the review and our foci.

### Stage 2: identifying relevant studies

#### Databases

The following databases were chosen as our primary sources for studies: Medline (via OVID), PsychINFO, PubMed, SCOPUS, and Web of Science. In addition, bibliographies of papers returned from the databases and deemed appropriate for this review were screened in order to identify any papers missed during the database searching process.

##### Search strategy

KP, AK, and MD developed the search strategy in collaboration with the department librarian. Here, we display a very simple outline of the search strategy we used to gather our studies (see Additional file 1). The full search strategy can be found as an additional file (see Additional file 2). As suggested by Daudt et al., we were flexible and iterative in the use of our search terms [21] and in our generation of the final search strategy. We developed this search strategy with the intention of capturing the widest possible selection of papers in our field of study. However, we acknowledge that some papers that are very specific in their terminology (i.e., referring to a specific disorder only by its specific name) may have been missed.

### Stage 3: study selection

Two reviewers (KP, CC) independently screened titles and abstracts of all the papers returned from the five databases. Titles and abstracts that were clearly irrelevant were discarded at this stage. Full texts of all remaining papers were accessed; a copy of any paper that was unavailable via the university library was requested from their author. Papers about the same study (i.e., different papers using the same dataset) were identified, and their contents were merged together. Next, KP and CC independently reviewed the full texts against inclusion and exclusion criteria as defined below; and two further reviewers (AK and MD) each reviewed a random selection of 10% of the papers that were reviewed by KP and CC. The review team started the process by reviewing together a small sample of studies in order to ensure that there was an agreed common understanding about the inclusion and exclusion criteria. Disagreements about papers were discussed midway, and at the end of the process, as per guidelines set out by Levac et al. [23]. Also, we ensured that one of the reviewers was not an expert in the field, so as to avoid any pre-formed bias. All references were organised and shared through the bibliographic software Mendeley.

#### Inclusion and exclusion criteria

As suggested by Armstrong et al. [20], inclusion and exclusion criteria were broad in order to maximise the number of relevant studies captured by the scoping review and to map comprehensively the breadth of literature. In order to do this, we started with core criteria and added to these criteria from our reading of abstracts. For example, mental health conditions were included in the first iteration of the criteria. After a first reading of the abstracts, these core criteria were then expanded to include studies involving suicide. We considered that this “broadening” approach was in keeping with the methodology of a scoping review as it lends itself to the eventual creation of criteria that provides comprehensive coverage and includes relevant aspects that may have been missed in the first screening (e.g., it is unlikely that studies involving suicide would have been captured using our initial criteria of “papers involving mental health conditions”). These criteria were informed by official definitions of key terms and discussion among the review team.

##### Inclusion criteria

- *Register-based studies*: Any study that was based on data obtained from registries, nationwide or otherwise, was included in the review.
- *First- and second-generation migrants*: All papers involving first-generation migrants were included. There is currently no universally recognised definition for the term “migrant.” In this study, we used a definition adapted from the International Organisation for Migration’s definition: “any person who is moving or has moved across an international border or within a State away from his/her habitual place of residence, regardless of (1) the person’s legal status; (2) whether the movement is voluntary or involuntary; (3) what the causes for the movement are; or (4) what the length of the stay is.” [24]. This definition was chosen as it has been used widely among other papers in this field, and we feel that it fully captures the criteria we attribute to the term. Papers that reported about first- and second-generation migrants (defined as the child of one or two migrant parents, born in the country that his/her parent(s) migrated to) were included if the two groups could be analysed separately and comparatively.
- *Papers involving refugees*: Papers that investigated populations of refugees, asylum seekers, or individuals living in exile were included in the review (in keeping with the UN definition of a migrant).
- *Mental health conditions*: All mental health conditions were included in the study. Examples include depression, anxiety, schizophrenia, borderline personality disorder, bipolar disorder, and learning disabilities. Non-clinically assessed outcomes (e.g., self-reported mental ill-health) were included.
- *Studies involving suicide*: Papers involving the study of suicide were included, due to the link between suicide mortality and mental disorders.

##### Exclusion criteria

- *Within-country migration*: Studies that dealt solely with migration within a single country were excluded.
- *Non-English language papers*: Due to lack of funding for the translation of non-English language papers, they were excluded from the review.

### Stage 4: charting the data

In accordance with the guidelines set out by Arksey and O’Malley [22] and supplemented by the Cochrane Handbook [25], the data reported in the eligible papers were charted in an Excel spreadsheet. This charting process provided an at-a-glance view of general information about the studies as well as specific information that was useful during the reporting and discussion of the results. The categories for this spreadsheet were informed largely by Armstrong et al. [20] and are defined in (see Additional file 3: Table S2).

### Stage 5: collating, summarising and reporting the results

Stage 5 presented an overview of all material by conducting a thematic analysis. This involved the following guidance from Braune and Clarke’s 2006 paper [26]. We took an inductive approach, letting the content of the included studies guide our theme development. Following the step-by-step guidelines (broadly: familiarisation with the data; the generation of initial codes; and searching, reviewing, and defining themes), we identified a highlighted certain patterns across the papers in our study in our charting exercise. We then conducted a numerical analysis. Simple numerical counts of each category were included in the charting exercise and this aided the generation of a narrative description of the research conducted in each study area. Sub-categories were established next, and a thematic analysis of studies within each category was conducted.

### Study quality assessment

Scoping reviews do not typically contain a quality assessment of studies. However, in light of arguments put forward suggesting that such an assessment would help improve rigor and interpretation, we decided to use guidelines to appraise the quality of data linkage studies developed by Bohensky et al. [27]. These guidelines are split into four major “domains,” each of which assess (1) the existing data sources to be linked; (2) researcher-selected variables and data preparation; (3) the linkage process; and (4) ethics, privacy, and data protection. KP applied the quality assessment guidelines to all included studies, and AK and MD each reviewed an independent random selection of 10% for cross-checking reliability.

## Results

All searches were carried out on 25 November 2015. The five databases used in the review were searched independently, and all returned records were compiled (*n* = 1309) before the duplicates were removed. Of the 916 remaining records, 805 were excluded during the initial screening processes, and a further 60 were excluded after the second screening. The remaining 51 papers were included in this review. A flow chart of this process can be found as (see Additional file 4: Figure S1).

Where available, the following information was extracted from each paper and charted in Additional file 5: Table S3: author; year of publication; study title; country of interest; migrant type, age range, and population size; registers used; main outcomes and findings; and documented advantages and disadvantages of using register data for research (see Additional file 5).

We used “vote-counting” rather than a quantitative synthesis to present our results in keeping with the general methodological approach of scoping reviews and to provide a clear scope of the topics that are researched in this area. In addition, a more robust meta-analysis method would have been inappropriate given the high level of heterogeneity of our studies. This method lends itself to certain limitations however. Comparing numbers of studies does not necessarily reflect comparison by the magnitude of the population, confidence in the impact, or concerns about risk of bias.

### Study quality

Six of the studies included in this paper were not linkage studies; they used only one register data source. As a result, those studies were not assessed for quality using Bohensky’s guidelines. In addition, as there is no need for ethical approval for most register-based studies in certain Nordic countries (e.g., in Finland), which make up the majority of our included studies, we did not assign scores for ethical approval (domain 5) for any study. Included studies used varying numbers of datasets in their research and that not every criterion was applicable to every study. Our scores were calculated based on the average number of points attained in each domain. This approach allowed us to “control” for the number of datasets that were used in a study. The point scores for each study can be seen in (see Additional file 6: Table S4).

The median point score and both quartiles for the studies were then calculated in order to identify “low,” “medium,” and “high” quality studies.

Additional file 7: Figure S2 graphs the point score for each of the record-linkage studies that were included in this review (see Additional file 7). We found that 11 studies achieved a score that met our criteria for a “high quality” study. Only two of those 11 studies were conducted outside of Nordic countries or the Netherlands. Interestingly, two of the oldest four papers in the review were also in this group of “high-quality studies.”

Three of these papers looked at incidence rates of schizophrenia and found that refugee status was a reliable predictor of risk among migrants. Selten’s 1997 study found that all migrant groups had a much higher risk of schizophrenia onset than the settled majority in the Netherlands [28], whilst Anderson’s 2015 study in Canada found that this was only the case for migrants from the Caribbean, whereas all other groups had lower risks [29]. Both high-quality papers studying autism found that children born to migrant parents had significantly lower risk of being diagnosed with an autism spectrum disorder [30, 31]. Wallach-Kildermoes’ study was the only high-quality study to look at medication use and found that migrants were less likely to initiate treatment after a medical facility discharge than the settled population [32].

Webb’s 2015 study in Denmark was the only high-quality study to look at suicide rates and found that the relative risk of attempted suicide for migrants was higher than that for the settled population, with higher rates for females than those for males [33]. Selten’s Dutch 1994 study found that hospitalisation for schizophrenia carried a statistically non-significant change in risk from that of the settled majority [34], whilst the later 2012 study found risk for unipolar depressive disorder was much higher for migrants [35]. Munk-Olsen’s Danish study found that perinatal migrant mothers had more contact with psychiatric services than the mothers from the settled population [36]. Youngmann’s Israeli study found that whilst migrants had more contact in the 1980s, the difference became smaller in the 1990s [37].

We also identified five low-quality studies. Interestingly, some of them had very different results to the high-quality studies. Lehti’s 2013 study in Finland found that children with two migrant parents had an increased likelihood of being diagnosed with autism [38], a direct opposite to the findings of the similar high-quality paper. Di Thiene’s 2015 study in Sweden concluded that migrants had a significantly lower risk of attempting suicide than the settled population [39]. Laubjerg’s study found that international adoptees have higher psychotic disorder rates than non-adoptees [40]. Mezuk found that migrants living in enclaves in Sweden did not have an increased risk of psychosis or affective disorders [41], and Lay’s 2007 study observed that migrant inpatients were significantly younger on average than the Swiss settled population [42].

### General findings

We identified studies from 14 different countries, over half of which (27/51; 53%) were conducted in Nordic countries (12 in Sweden, 10 in Denmark, 3 in Norway, and 2 in Finland). The Netherlands (6/51; 12%), Israel (5/51; 10%), and Canada (4/51; 8%) make up a further 19 studies, whilst no more than 3 studies were conducted in any one other country.

The majority (32/51; 63%) of identified studies focused on first-generation migrants. Of these, 12 concentrated on refugees and/or asylum seekers (7/32; 22%). A further eight studies focused on second-generation migrants (10/51; 20%), of which two focused on inter-country adoptees (2/10; 20%). The remaining nine papers did not differentiate between first- and second-generation migrants, or studied both (9/51; 18%).

Our charting exercise revealed four major branches of research within the topic of migrant mental health: rates and risks of psychiatric disorders, rates and risks of suicide mortality, the use of psychotropic drugs, and health service utilisation and mental health-related hospitalisation rates.

#### Rates and risks of psychiatric disorders

Nineteen of the identified studies (19/51; 37%) looked at rates and risks of psychotic disorders as a measure of migrant mental health. Twelve of these studies identified a higher relative risk of developing a psychotic disorder for migrants than for the settled majority (12/19; 63%). Two studies (2/19; 11%) identified no statistically significant difference between the two groups, one of which was an Israeli study in which the incidence of schizophrenia in second-generation migrants was no different to that of the settled majority [15]. A further four studies (4/19; 21%) found that migrants had a lower recurrence-risk of developing a psychotic disorder than the settled majority. One found migrants could have either a higher or lower risk in the studied country depending on the migrant’s country of birth (1/51; 5%) [29].

These differences become clearer when the studies are categorised in terms of the specific psychotic disorders studied. Of the 19 papers, 10 focused on schizophrenia (10/19; 53%), 4 focused on autism spectrum disorders (4/19; 21%), 1 for each of bipolar (1/19; 5%) and dementia (1/22; 5%), and two that looked at psychotic disorders in general (2/19; 11%).

##### Schizophrenia

Of the studies focusing on schizophrenia, the majority (8/10; 80%) found that migrants have a higher risk of developing schizophrenia than the settled majority. One of these studies found higher rates of psychotic disorders among migrants moving from the Caribbean/Bermuda to Canada (incidence rate ratio (IRR) 1.60, 95% CI 1.29–1.98) but noted much lower rates for those migrating from Europe and East Asia [29].

##### Bipolar disorder

Selten’s 2003 study focused on bipolar disorder and found that, whilst there is no difference between migrants and the settled majority in terms of the risk of developing manic or circular type bipolar disorder, migrants have a significantly higher risk of depressed type bipolar disorder [43].

##### Dementia

Diaz’s 2015 paper suggests migration is negatively correlated with risk of developing dementia; a significantly lower proportion of migrants had a diagnosis of dementia when compared to the settled majority [44].

##### Autism spectrum disorders

Two papers found that second-generation migrants had an increased likelihood of being diagnosed with autism spectrum disorders. For example, Lehti’s study compared children of migrants to children with two Finnish parents and found that risk of autism was increased (adjusted OR 1.8, 95% CI 1.2–2.7) [38]. However, a further two papers found otherwise, including Van der Ven’s study that found children born to migrants from developing countries were at lower risk of autism spectrum disorder than children born to Dutch parents (RR = 0.6; 95% CI 0.5–0.9) [45].

#### Different migrant types

##### Adoptees

Both studies that focused on inter-country adoptees found increased risks of developing psychotic disorders (2/2; 100%). One of these studies, conducted by Cantor-Graae in Denmark, showed that the relative risk of developing schizophrenia as an inter-country adoptee with two foreign-born parents was 2.9 (95% CI 2.4–3.5) [46].

##### Refugees

Studies comparing the mental health of refugees to settled populations found that refugees had higher rates of risk (2/2; 100%). A study by Anderson showed rates were especially high for refugees moving from East Africa (IRR = 1.95, 95% CI 1.44–2.12) and South Asia (IRR = 1.51, 95% CI 1.08–2.12) to Canada [29]. The study conducted by Hollander in Sweden found that refugee migrants also had an increased risk of developing a psychotic disorder than other non-refugee migrants, with a significant difference present between female migrants within the two groups (OR = 1.27, 95% CI 1.15–1.40) [47].

#### Rates and risks of suicide mortality

Of the 11 papers (11/51; 22%) that focused on suicide mortality, 10 compared rates and risk for migrants with the settled majority population (10/11; 90%). Three found that either the suicide death rates or suicide risk rates were higher among all migrant groups studied than in settled majority populations (3/10; 30%). One of these studies showed that people who migrated from South Asia had a twofold higher risk ratio than the settled population in Brazil (RR = 2.99; 95% CI 1.06–4.34) [48]. Four other studies (4/10; 40%), however, found that the migrant population’s rates and risks were lower than the settled majority. For example, Pavlovic’s study in Australia found that Croatian migrants had a much lower suicide rate (3.10/100,000/year) compared to the Australian settled majority population (13.06/100,000) [49]. The remaining three studies (3/10; 30%) found that the risk of suicide mortality for migrants was significantly variable depending upon other factors, such as age, gender, or country of birth. The only study in this review carried out in Hong Kong found that short-duration adolescent migrants had much lower suicidality levels than Hong Kong-born adolescents [50].

Six papers (6/11; 55%) assessed the risks and rates of suicide mortality for second-generation migrants. Of these, five (5/6; 83%) found that the risks were higher for migrants than those for the settled majority, though not always quite as high as the risks for first-generation migrants.

The one paper (1/11; 9%) that made comparisons between different generations of migrants found that first-generation migrants had a lower suicide risk than second-generation migrants, who in turn had a lower suicide risk than third-generation migrants [51].

#### The use of psychotropic drugs

All four (4/51; 8%) of the papers that studied the use of psychotropic drugs among migrants focused on first-generation migrants. Two (2/4; 50%) of these found that the rates of use of psychotropic drugs for all studied migrant groups were significantly lower than those for the settled majority. For example, a Danish study found that migrants had higher odds of not initiating treatment for Alzheimer’s disease than the settled population (OR = 1.55; 95% CI 1.01–2.38) [32].

Hollander’s 2011 study found that refugees were more likely to purchase psychotropic drugs than non-refugee migrants, but specified that this increased likelihood was for women only (OR = 1.27 95% CI 1.15–1.40) [52].

Two studies (2/4; 50%) looked specifically at refugees. One of these (1/2; 50%) found that migrants were less likely to use psychotropic drugs than the settled majority population [53]. Again, the study that disagreed only found that the reverse was true for women [52].

#### Health service utilisation and mental health-related hospitalisation rates

Seventeen of the included studies (17/51; 33%) focused on the frequency of contact that migrants had with mental health services. Eight studies (8/17; 47%) found that migrants had higher contact rates than settled majority populations, whereas ten (10/17; 59%) found that their contact rates were lower.

This becomes clear when the studies are categorised by the type of contact populations had with mental health services. Of the eleven studies (11/17; 65%) that focused on voluntary health service use, eight (8/11; 73%) found that migrants were less likely to utilise health services than the settled majority. Two of the three studies (3/11; 27%) that found the reverse was true were carried out in Denmark, with the other carried out in the Netherlands. The two studies (2/17; 12%) that looked specifically at refugees had different results. Weinstein’s study in the USA found that refugees were infrequent users of health services [54], whilst Norredam’s study in Denmark found that both refugee males (RR = 2.02; 95% CI 1.75–2.34) and refugee females (RR = 1.49; 95% CI 1.29–1.72) had a higher risk of psychiatric contact that the settled majority [12].

Six studies (6/17; 35%) focused on involuntary hospitalisations, and five (5/6; 83.3%) found that rates for migrants were higher compared to settled populations. For example, a Swedish study found that among those who had become unemployed, migrant females had a much higher risk (Hazard Ratio = 3.47, 95% CI 3.02–3.98) of hospitalisation than the general population [55]. The only study (1/6; 17%) that found that migrants had lower rates of mental health-related hospitalisation was carried out in Switzerland. Lay’s 2007 study found that among immigrants, the proportion of female inpatients (39%) was much lower than in the general population (46%) [42].

## Discussion

Scoping all published papers in this research area identified 51 independent studies. More than half of these studies were carried out in Nordic countries, probably due to the long-standing tradition these countries have of collecting data for health registers as well as a high public acceptability of register data use. Between the years of 1943 and 2007, the Nordic countries of Denmark, Finland, Iceland, Norway, and Sweden started nationwide health registers for each of the following topics: hospital discharges/inpatient care, hospital outpatients/ambulatory care, birth, cancer, prescription, and causes of death. In addition, these registers use national identification numbers which makes it possible to link multiple datasets easily [5].

Whilst many more studies in our review focused on first- rather than second-generation migrants, the distribution of studies between countries was very similar for both. The studies focusing on refugees were also distributed fairly evenly, whilst both studies looking specifically at inter-country adoptees were carried out in Denmark [10, 25].

Our results showed that studies analysing psychiatric disorders produced very varied results. This variation was observed between studies and also across psychiatric disorders.

Our analysis showed that refugee migrants had worse mental health than non-refugee migrants in general. This finding may be due to the forced nature of their migration as well as traumatic experiences they may have had prior to migration. These migration experiences are likely to include exposure to a number of situations that can have adverse impacts on mental health. Violence, abuse, uncertainty, and trauma are common factors in many refugee migration experiences. In addition, the migration process itself has been shown to be a traumatic event. This process, with a focus on those who migrate as refugees, has been covered widely in the media and may also play a part in their mental health post-migration. The socio-demographic profile of refugees is also likely to be different to economic migrants. Factors such as age, education, ethnicity, and physical health status are important determinants of mental health.

Whilst migration has been repeatedly found to be associated with higher suicide mortality [48, 49], interestingly, the only study to compare multiple generations of migrants found that risks were also positively correlated with increasing migrant generations. This goes some way to supporting a degradation of the healthy migrant effect over time; mental health has declined among migrants over the course of generations [38]. This variation may be explained by cultural differences. It is possible that either higher or lower suicide rates for migrants in a new country are not an accurate predictor of mental health, and that instead, the rates are largely dependent upon the difference between the cultural and religious views of suicide within a migrant group and the settled population.

There was a large amount of variance in the rates of suicide mortality of migrants between different countries. Whereas migrants from South Asia have been shown to have a much larger risk of suicide mortality than settled majority population [29, 48], Croatian migrants in Australia had a much lower risk [49]. This variance may have as much to do with the original country of the migrants than the new, hosting country, and should be researched further.

The use of psychotropic medication was only studied for first-generation migrants. However, there is some evidence showing that especially second-generation migrants have a high prevalence of mental health problems [10, 38]. Second-generation migrants need to be studied to expand upon this area of register research. For those studied, male migrants tended to have a lower rate of psychotropic medication use, whereas female migrants tended to have a higher rate [46].

Migrants were found to use mental health services less often than the settled majority in most studied countries. This is not true, however, for migrants in Denmark [12] and the Netherlands [54], and the reasons for this could form the basis of further research. Whilst it would seem likely that refugees are less likely to make use of health services than settled majority populations, the evidence is different, and further research needs to be conducted. Hospitalisation rates were increased for migrants in most countries, the only exception being in Switzerland [42]. An assessment on the procedures in Switzerland might help to explain this anomaly.

### Advantages and disadvantages of using register data in migrant mental health research

The biggest and most widely recognised advantage of using register-based health data is the larger, often nationwide, sample size. This leads to high external validity; conclusions reached for such large samples can often be generalised for the entire populations with high confidence [56, 57]. For those data covering entire populations, the risk of selective attrition is eradicated [53]. Large sample sizes also reduce the risk of type one errors, i.e., the false rejection of a null hypothesis [47]. In addition, larger samples allow for the categorisation and subsequent analysis of sub-groups within a population [58], such as different migrant groups.

The quality of data within health registers is also generally relatively high [6]. Systems in place within individual countries also make it likely that the databases used are highly complete. For example, there are mandatory childhood development assessments in Stockholm, which makes it very likely that a large proportion of those suffering from autism would be diagnosed, and their data are available in health registers [31]. Diagnoses taken from health data were entered by physicians, and so are more valid than self-reported surveys [12]. The effect of incomplete registers may be minimised via the linking of separate databases to overcome some of the weaknesses of each database [6]. Register-based studies have high-concordance with survey-based studies [59]; and finally, the low cost of register-based studies is an advantage [59].

However, register-based studies do have certain disadvantages. There are a number of ways in which completeness or quality of registers may be compromised and affect the validity of the studies that make use of them. For example, the information on medication within prescription databases will include data on medication purchased in the “host” country; any medication bought from other countries or online will go unrecorded [53]. Prescription registers typically do not record the indication for the prescribed medication; some psychotropic drugs are commonly prescribed for other indications than mental disorders. Therefore, we cannot say with confidence that psychotropic drug use automatically indicates the existence of mental disorder. Whilst physician diagnoses are very useful, misdiagnosis can occur, which will affect the quality of the associated register [47]. In addition, discrepancies in the coding of disorders between physicians are common. In terms of suicide mortality, cultural factors may mean that cases of suicide will be unreported or coded as something other than suicide [13]. Similar cultural factors, such as stigmatisation of mental disorders in a migrant’s home country will influence the rates at which medical services are accessed [3]; the under-use of these services may lower the validity of register data as an indicator of mental health problems. The under-reporting of emigration to a second “host” country is also fairly common and leads to an overestimation of migrant population sizes [60]. Register studies are also restricted by the health data that is recorded. Important background information for migrants, such as reasons for migration, length of stay, or health behavior data are typically not reported in health registers [54, 61].

### Limitations

This review allowed us to develop an overview of the available literature in the field. The use of scoping review methodology was particularly advantageous as we were not restricted to tight inclusion criteria, and we could present our results in an accessible format. We were also able to easily identify the gaps in the current literature, which allows for the easy generation of new research questions. However, we were limited in a number of ways. We were unable to use a more robust meta-analysis to sum and synthesis the results from each study. A quantitative synthesis may have revealed additional insights. We were unable to fully answer our subsidiary questions, due to the limitations of our search strategy. Not encompassing all studies using data other than registry data hindered our ability to fully analyse the effectiveness of registry studies in comparison to other study types.

### Further research

Further research should be carried out in this subject area, specifically within the domains that are under-researched (e.g., use of psychotropic drugs and refugees). Of course, the availability and quality of data varies from country to country, but we have identified a lack of register studies within Africa and very few within South America. Although 37% of the papers in the study looked at incidence rates of mental disorders, bipolar disorder and dementia remain under-represented; the disorders were the subject of one paper each. Only 8% of studies looked at the use of psychotropic drugs, and so this area should be explored further. Whilst 7% of studies looked at the mental health of refugees, further studies are needed especially in light of the recent surge of refugee migration.

## Conclusions

This study is the first of its kind to scope the current body of literature concerning the mental health of migrants, as informed by register-based studies. We offered a unique insight into the ability of register studies to inform us about migrant mental health, finding that migrants generally have an increased risk of both developing mental health disorders and suicide mortality, whilst they utilise mental health services and psychotropic medication less than the settled majority population. We also find that for each of the four domains in which refugee mental health is studied, it is generally poorer than that of non-refugee migrants. However, we acknowledge that there is much variation within and between these categories. We also evaluate the effectiveness of registry-based studies of analysing the mental health of migrants by highlighting reported advantages and disadvantages of individual studies, and the feasibility of conducting further reviews on more specific sections of this study area.

## Additional files

Additional file 1: Overview of search strategy. A brief overview of the terms used in our search strategy. Full search strategy can be found as Additional file 2. (DOCX 30 kb)

Additional file 2: Full search strategy. Our full search strategy for finding relevant studies in our database searches. (DOCX 21 kb)

Additional file 3: Data extraction form. Defining the variables we would be extracting from each individual paper in the charting exercise. (DOCX 14 kb)

Additional file 4: Figure S1. Outlining the exclusion process: from initial database searches to studies included in the review. A flowchart to show the numbers of studies making it through each stage of our study selection process, from initial searches to studies included in qualitative synthesis. (DOC 21 kb)

Additional file 5: Table S3. Charting key data for each study included in this review. A table charting the following data for each study in this review: author; year of publication; study title; country of interest; migrant type, age range, and population size; registers used; main outcomes and findings; and documented advantages and disadvantages of using register data for research [10, 12, 15, 16, 19, 27–54, 56–58, 60–74]. (XLSX 27 kb)

Additional file 6: Table S4. Appraising each included study using the guidelines set out by Bohensky et al. Description of data: Study number corresponds to the number assigned as “Quality Assessment Number” in Additional file 5: Table S3. “1” indicates criterion is fulfilled; “0” indicates criterion is not fulfilled; blank space indicates criterion is not applicable. (XLSX 20 kb)

Additional file 7: Figure S2. Graphing the studies included in the review by their scores against Bohensky’s quality assessment criteria. Study number corresponds to the number assigned as “Quality Assessment Number” in Additional file 5: Table S3. (DOCX 46 kb)
