# Supplementary material for: What do register-based studies tell us about migrant mental health? A scoping review
Source: Syst Rev. 2017 Apr 11;6:78. doi: 10.1186/s13643-017-0463-1 (PMC5387245; doi:10.1186/s13643-017-0463-1)
Supplement: Supplementary file 2 — Full search strategy. Our full search strategy for finding relevant studies in our database searches. (DOCX 21 kb) [file 13643_2017_463_MOESM2_ESM.docx]

**Screening**

**Included**

**Eligibility**

**Identification**

Records identified through database searching
(n = 1309)

Additional records identified through other sources
(n = 0)

Records after duplicates removed
(n = 916)

Records screened
(n = 916)

Records excluded
(n = 805)

Full-text articles assessed for eligibility
(n =111)

Full-text articles excluded, with reasons
(n = 60)

No focus on mental health (n=3)

No focus of migration (n=11)

Only within-country migration (n=7)

Not a record linkage study (n=31)

Not an English language paper (n=8)

Studies included in qualitative synthesis
(n = 51)

Studies included in quantitative synthesis (meta-analysis)
(n = N/A)
