# Supplementary figures and images for: What do register-based studies tell us about migrant mental health? A scoping review
Source: Syst Rev. 2017 Apr 11;6:78. doi: 10.1186/s13643-017-0463-1 (PMC5387245; doi:10.1186/s13643-017-0463-1)

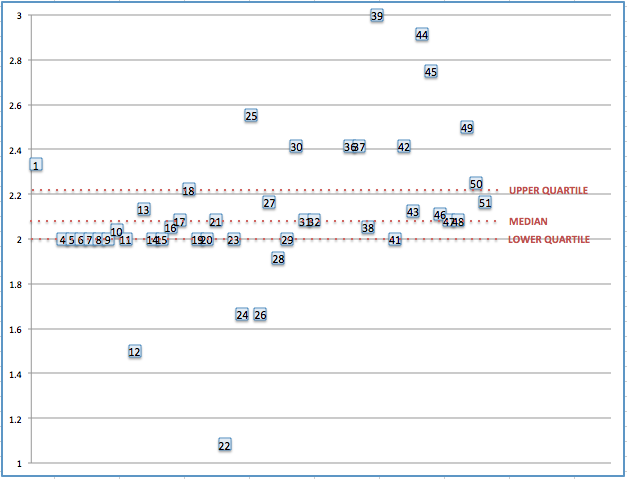

Supplement: Supplementary file 4 — Outlining the exclusion process: from initial database searches to studies included in the review. A flowchart to show the numbers of studies making it through each stage of our study selection process, from initial searches to studies included in qualitative synthesis. (DOC 21 kb) [file 13643_2017_463_MOESM4_ESM.doc]
