# Supplementary material for: What do register-based studies tell us about migrant mental health? A scoping review
Source: Syst Rev. 2017 Apr 11;6:78. doi: 10.1186/s13643-017-0463-1 (PMC5387245; doi:10.1186/s13643-017-0463-1)
Supplement: Supplementary file 7 — Graphing the studies included in the review by their scores against Bohensky’s quality assessment criteria. Study number corresponds to the number assigned as “Quality Assessment Number” in Additional file 5: Table S3. (DOCX 46 kb) [file 13643_2017_463_MOESM7_ESM.docx]

1 exp registries/

2 register based

3 regist$ adj2 (study OR data)

4 routin$ adj3 data

5 exp medical record linkage/

6 record-?link$

7 administrative data

8 OR/1-7

9 exp transients and migrants/

10 exp refugees/

11 exp ethnic groups/

12 Immigrant

13 Emigrant

14 asylum seekers

15 OR/9-14

16 exp mental health/

17 exp mental disorders/

18 exp behavior and behavior mechanisms/

19 mental adj2 (health OR illness$ OR disorder$)

20 psych$

21 bipolar.ti.ab

22 anxiety.ti.ab

23 suicid$.ti.ab

24 anti-?depress$

25 OR/16-24

26 8 AND 15 AND 25
